# *Cyclospora cayetanensis* infection in transplant traveller: a case report of outbreak

**DOI:** 10.1186/s13071-015-1026-8

**Published:** 2015-08-07

**Authors:** Małgorzata Bednarska, Anna Bajer, Renata Welc-Falęciak, Andrzej Pawełas

**Affiliations:** Department of Parasitology, Faculty of Biology, University of Warsaw, Miecznikowa Street, 02-096 Warsaw, Poland; Department of Gastroenterology, Hepatology and Clinical Oncology, Medical Center for Postgraduate Education, Roentgena Street 5, 02-781 Warsaw, Poland

## Abstract

**Background:**

*Cyclospora cayetanensis* is a protozoan parasite causing intestinal infections. A prolonged course of infection is often observed in immunocompromised individuals. In Europe, less than 100 cases of *C. cayetanensis* infection have been reported to date, almost all of which being diagnosed in individuals after travelling abroad.

**Findings:**

We described cases of three businessmen who developed acute traveller’s diarrhoea after they returned to Poland from Indonesia. One of the travellers was a renal transplant recipient having ongoing immunosuppressive treatment. In each case, acute and prolonged diarrhoea and other intestinal disorders occurred. Oocysts of *C. cayetanensis* were identified in faecal smears of two of the travellers (one immunosuppressed and one immunocompetent). Diagnosis was confirmed by the successful amplification of parasite DNA (18S rDNA). A co-infection with *Blastocystis hominis* was identified in the immunocompetent man.

**Conclusions:**

Infection of *C. cayetanensis* shall be considered as the cause of prolonged acute diarrhoea in immunocompromised patients returning from endemic regions.

## Findings

*Cyclospora cayetanenis* is a human parasite transmitted through the faecal-oral route which infects the small intestine [1, 2]. Fresh fruits, herbs and vegetables (raspberries, blackberries, basil, lettuce) are foods most commonly identified as a source of human infection [3–7]. *Cyclospora cayetanensis* has also been responsible for a few waterborne outbreaks in North America and elsewhere [8, 9].

Most *Cyclospora* infections have been reported in travellers (traveller’s diarrhoea) and in inhabitants of endemic areas such as Haiti, Guatemala, Peru and Nepal and also in the United States, Central America, South Asia and Eastern Europe [10–12]. Cases of *C. cayetanensis* infections in Europe are sporadic (less than 100 cases), and almost all were described in persons returning from endemic areas [12]. To date, no cases of *C. cayetanensis* infections have been reported in Poland. Reports on *Cyclospora* infections in organ transplant recipients are very rare and only 2 cases have been described in renal transplant recipients [13, 14]. The course of *Cyclospora* infections depends on the immunological status of the infected individuals. Cyclosporiasis is more severe in children and immunosupressed individuals, i.e., HIV/AIDS patients [15–17].

In this paper, an outbreak of cyclosporiasis in three travellers, including one renal transplant recipient, returning from Indonesia is described.

### Case presentation

Three businessmen from Poland developed acute diarrhoea after returning from a business trip to Indonesia in November 2013. They spent two weeks in Indonesia, travelling together and staying in various hotels, whilst visiting different areas of the country. Although they usually ate in a hotel restaurant and drank bottled water, they did occasionally consume regional food such as semi-dried meat, vegetable dishes and water served with ice, originating from unknown sources. Within 5 to 14 days after their return to Poland, acute diarrhoea and other intestinal disorders appeared in all three men. The course of the disease was different in each traveller, depending on their immunological status. The study was conducted under the project titled “The risk of opportunistic infections caused by parasites in men with immunodeficiency”. The project including the study on human fecal samples was approved by the Ministry of Science and Higher Education (Grant No. NN404101036)

#### Patient 1

A 35-year old man who received a renal transplant (RT) due to chronic glomerulonephritis and agenesis of the left kidney in November 2010. Since that time he has undergone immunosuppressive treatment, consisting of tacrolimus 0.5 mg twice daily, mycophenolic acid 360 mg once daily and deflazacort 3 mg twice daily. His medical history revealed sigmoid diverticulitis in September 2013, which was treated with metronidazole and ciprofloxacin. Two weeks after returning to Poland, he experienced acute diarrhoea with a minimum of ten bowel movements daily. He also developed flatulence, abdominal cramping and a general feeling of malaise. Intensive diarrhoea did not recede and within three months (November 2013 – February 2014) the patient lost 15 kg of his body weight. Within the same three-month period, the patient was hospitalised twice (in December 2013 and January 2014) because of dehydration. Due to significant decrease in body mass, his immunosuppressive treatment with mycophenolic acid had to be reduced (dose unknown). In December 2013, a colonoscopy was performed in this patient, with a biopsy taken from the terminal ileum showed histopathological abnormalities suggesting intracellular parasites (data from the interview). He was treated against coccidia/microsporidia infections with ciprofloxacin (2x500 mg) and metronidazole (3x500 mg), followed by vancomycin (4x125 mg). The treatment was unsuccessful and acute diarrhoea continued.

#### Patient 2

A 39-year-old immunocompetent (IMC) man had a 2-week period of persistent diarrhoea, with eight to nine daily bowel movements, that started about 7 days after he returned to Poland. The patient also developed a low-grade fever, fatigue, abdominal pain, bloating and large intestinal motility. His stools were watery, without blood or mucus. The patient did not seek medical advice and the symptoms resolved. However, in January 2014 there was a reoccurrence of diarrhoeal disease, with signs of blood in the discharge. The appearance of blood was related to another health problem, haemorrhoids, as reported by the patient. The symptoms of diarrhoea disappeared after the administration of anti-haemorrhoid drugs.

#### Patient 3

An immunocompetent man had a four-week period of acute gastrointestinal disease. Symptoms had begun two weeks after his return to Poland, similarly to the other two travellers. Accordingly to reports from the two other patients, the man suffered from the most severe onset of symptoms and was absent from work for several weeks. However, this man did not seek medical advice and no faecal samples were provided for the diagnosis of intestinal infections. As the infection of *C. cayetanensis* was not confirmed in this case, we treat this case as a third probable but not confirmed case of travellers’ cyclosporiasis.

### Methods and results

Fecal samples from the case 1 (RT) and 2 (IMC) were collected in February and March 2014 and were studied by three detection techniques for the diagnosis of the intestinal infections. Faecal smears were stained with the Ziehl-Nelsen technique and screened using light microscopy. Indirect immunofluorescence assay (IFA) [18] was performed for the diagnosis of *Cryptosporidium* and *Giardia* infections (Merifluor *Cryptosporidium*/*Giardia * kit, Meridian Diagnostics, USA). Additionally, to confirm preliminary diagnosis of *Cyclospora* infection, PCR amplification of the 18S rRNA gene fragment was conducted.

**Fig. 1:**
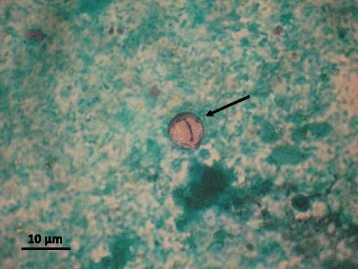
Oocyst of *Cyclospora cayetanensis* found in fecal smears of our RT patient.

Oocysts of *C. cayetanensis* were found in the faecal smears (Fig. 1) from both patients. From 5 to 20 *Cyclospora* oocysts per slide were found in the case of RT patient and 1–3 per slide in the case of the IMC patient. No oo(cysts) of *Cryptosporidium* or *Giardia* were detected in the IFA test.

Two nested PCRs were used to amplify the 18S rRNA gene fragments of *C. cayetanensis* [19, 20]. Sequencing of nested-PCR products confirmed the infection with *C. cayetanesis* (100 % homology with reference sequence from GenBank KC662292.1) (http://www.ncbi.nlm.nih.gov/genbank/index.html). Obtained 18S rDNA sequences of *C. cayetanensis* from both patients (case 1 and 2) were deposited in the GenBank database under the accession numbers KP642664 and KP642665, respectively. Sequencing of PCR products also revealed the presence of *Blastocystis hominis* and *Debaromyces hansenii* DNA in IMC patient. ITS1 sequences of *B. hominis* from the IMC patient was deposited in the GenBank database under the accession number KP675947. No further samples were available for diagnosis and the ICM patient did not start the treatment.

Following the diagnosis of a *Cyclospora* infection, the RT patient was treated with two daily doses of orally administered trimethoprim–sulfamethoxazole (960 mg) for a period of 10 days. Diarrhoea symptoms began to subside gradually from the 5th day and resolved after 8 days from the start of the treatment. Furthermore, the loss of patient’s body weight stopped. A control study conducted 3 months later by microscopic and PCR methods did not detect *Cyclospora* oocysts/ DNA in three independent stool samples.

## Discussion

In this report, we present the first outbreak of *C. cayetanensis* infections in three men from Poland, who developed acute diarrhoea after travelling to Indonesia. Prolonged watery diarrhoea is the main clinical manifestation of most infections with *C. cayetanensis* [2, 11]. This symptom was also observed in patients described in our study. The diarrhoea was the most severe and lasted as long as three months in the renal transplant recipient. In our report, the source of invasion was probably the same in each case, given that the symptoms occurred almost simultaneously. Three days before their return to Poland they had a meal in a local restaurant, where they drank non bottled water. This seems to be the most likely reason for the invasion of *C. cayetanensis*. Current literature describes nine waterborne outbreaks of *C. caytetanensis* [8, 9]. In these cases the number of the infected persons ranged from only a few to several dozen people, and the smallest described was a family outbreak which affected only 3 individuals [21].

Host susceptibility seems to be the most important factor that influences the course of the cyclosporiasis. A similar course of the disease has been reported in two immunocompetent men. The diarrhoea and other intestinal disorders subsided within a period of 2–4 weeks in both cases. The prolonged, persistent diarrhoea with significant weight loss occurred only in the renal transplant recipient, which resulted in dehydration and subsequent hospitalisation.

Knowledge on *Cyclospora* infections in patients after solid organ transplantation is limited. To our knowledge, only two cases of cyclosporiasis have been recently described in renal transplant recipients [13, 14]. This case of cyclosporiasis in a renal transplant recipient is the first reported from central Europe and Poland.

In our study, the primary diagnosis and treatment applied for the transplant recipient were incorrect. *Cyclospora* is an unknown pathogen in Poland. The routine stool examination for parasite cysts or ova may not be appropriate to detect the oocysts of the *Cyclospora* species. This was probably the main reason for the delayed diagnosis in the case of the RT patient. The treatment with trimethoprim and sulfamethoxazole, recommended by CDC for *Cyclospora* infection (http://www.cdc.gov/parasites/cyclosporiasis/treatment.html), was used successfully in previously diagnosed cases, leading to full recovery (22, 25) The use of appropriate drugs appears to be necessary for an effective treatment of cyclosporiasis in immunodeficient patients, such as transplant recipients, HIV-positive individuals, or in those under oncological or steroid treatment [11, 22–24]. In our study, we observed the resolution of symptoms in two immunocompetent men without any medical treatment. However, the secondary onset of diarrhoea, likely to persisting parasite invasion, and then prolonged asymptomatic infection was noted in one of them (case 2).

### Conclusion

Detection of *Cyclospora* infection may be problematic in non endemic countries of central Europe but accurate identification of the parasite species may facilitate rapid recovery. Infection of *C. cayetanensis* shall be considered as the cause of prolonged acute diarrhoea in immunocompromised patients returning from endemic regions.

#### Ethical approval

Written informed consents were obtained from the patients for publication of this case report.
